# Genetic Variation in Safflower (*Carthamus tinctorious* L.) for Seed Quality-Related Traits and Inter-Simple Sequence Repeat (ISSR) Markers

**DOI:** 10.3390/ijms12042664

**Published:** 2011-04-19

**Authors:** Pooran Golkar, Ahmad Arzani, Abdolmajid M. Rezaei

**Affiliations:** 1 Department of Agronomy and Plant Breeding, College of Agriculture, Shahid Bahonar University of Kerman, Kerman 76169133, Iran; 2 Department of Agronomy and Plant Breeding, College of Agriculture, Isfahan University of Technology, Isfahan 8415683111, Iran; E-Mails: a_arzani@cc.iut.ac.ir (A.A.); am.rezai@cc.iut.ac.ir (A.M.R.)

**Keywords:** genetic diversity, biochemical, fatty acids, marker, safflower

## Abstract

Safflower (*Carthamus tinctoriou*s L.) is an oilseed crop that is valued as a source of high quality vegetable oil. The genetic diversity of 16 safflower genotypes originated from different geographical regions of Iran and some with exotic origin were evaluated. Eight different seed quality-related traits including fatty acid composition of seed oil (stearic acid, palmitic acid, oleic acid and linoleic acid), the contents of, oil, protein, fiber and ash in its seeds, as well as 20 inter-simple sequence repeat (ISSR) polymorphic primers were used in this study. Analysis of variance showed significant variation in genotypes for the seed quality-related traits. Based on ISSR markers, a total of 204 bands were amplified and 149 bands (about 70%) of these were polymorphic. Cluster analysis based on either biochemical or molecular markers classified the genotypes into four groups, showing some similarities between molecular and biochemical markers for evaluated genotypes. A logical similarity between the genotype clusters based on molecular data with their geographical origins was observed.

## 1. Introduction

Safflower (*Carthamus tinctorius* L.) is believed to have been domesticated somewhere in the Fertile Crescent region over 4000 years ago [1,2]. Knowles [2], proposed seven diversity centers for safflower germplasm evolution including the Far East, India-Pakistan, the Middle East, Egypt, Sudan, Ethiopia and Europe. The Middle East center was sub-divided into three gene pools of Iran-Afghanistan, Israel-Jordan-Iraq-Syria, and Turkey [1]. Recently, the Near Eastern origin of safflower was supported by Chapman *et al.* [3]. Safflower lines native to each “center” are considerably similar in height, branching, spines, flower color and head size, whereas consistent morphological variations are retained between the centers [4]. Normal types of the whole seed contain 27–32% oil, 5–8% moisture, 14–15% protein, 2–7% ash, and 32–34% crude fiber [5,6]. Safflower is one of the crops with the greatest variability of fatty acid in its seed oil composition [7,8]. Safflower oil contains the saturated fatty acids of palmitic (C16:0) and stearic (C18:0) and the unsaturated fatty acids of oleic (C18:1), linoleic (C18:2) and linolenic (C18:3) [8,9]. Conventional safflower seed oil has a fatty acid profile made up of 6–8% palmitic acid, 2–3% stearic acid, 16–20% oleic acid, and 71–75% linoleic acid [10].

The efficiency of assessment of genetic diversity to be used in a breeding program will be improved if combined biochemical and molecular marker data are used [11,12]. Safflower genotypes have indicated considerable diversity across different geographical regions of the world [2]. The fatty acid composition of seed oil varies remarkably both between and within species, with fatty acids altering in both chain length and degrees of desaturation. Genetic variation for fatty acid composition is vital for genetic improvement of the oil quality in oilseed crops [10].

Various markers—morphological, biochemical, and molecular—are used to assess plant genetic diversity. With the advent of DNA markers, possessing the advantages of higher polymorphism and independent of environment and plant growth stage, they have been widely employed for the assessment of genetic diversity [13]. Inter-simple sequence repeat (ISSR) is a DNA based marker with primers designed based upon dinucleotide, tetranucleotide or pentanucleotide repeats [14]. ISSR markers, with the advantages of simplicity, acceptable stability and high reproducibility, have been successfully used in genetic variation studies, gene mapping, germplasm identification and fingerprinting construction [12,15,16]. ISSR markers are more specific than RAPD markers, because of their longer SSR-based primers with higher primer annealing temperature, enabling amplifications of more reproducible bands [17]. The ability to reveal genetic variation among different genotypes may be more directly related to the number of polymorphisms detected with each marker technique rather than a function of which technique is employed [18]. Genetic variation in safflower has been studied using agro-morphological traits [19–21], biochemical traits in seed [22,23] and molecular markers including EST-SSR [24] AFLP [25], ISSR [26–28], and RAPD [29,30]. Moreover, genetic purity of safflower hybrids was estimated using EST-SSR markers in safflower [31]

Although, molecular markers have already been used either alone [25–27], or together with agro-morphological traits [28,30], to assess the genetic diversity in safflower, the relationships between molecular markers and seed quality-related traits are lacking in this oilseed crop. The objectives of this study were to assess the genetic variation of C. *tinctorius* genotypes with native and exotic origins using molecular markers and seed quality-related biochemical traits and to compare the results obtained by these two methods.

## 2. Results and Discussion

### 2.1. Seed Quality-Related Traits

The results of analysis of variance showed a significant difference among safflower genotypes for protein, oil, ash, fiber and fatty acid contents of seed (Table 1).

Seed oil content of the genotypes ranged from 21% in Wht-ISF to 33.5% in Mex.2-138 (Table 2). Regarding protein content, K_21_ (25.6%) and Mex.13-216 (13.5%) possessed the highest and the lowest protein content, respectively.

Palmitic acid of safflower genotypes ranged from 6.49% in Mex.13-216 to 11.07% in ISF_14_ (Table 3). Mean of stearic acid content (%) varied from 1.43 in Mex.13-216 to 2.94 in GE_62918_ genotype (Table 2). With considering the breeding aims in decreasing these two saturated fatty acids, using Mex.13-216 genotype could be recommended. Oleic acid content showed the highest variation in the studied genotypes. Oleic acid ranged from 14.1 (ISF_14_) to 35.26% (Mex.22-191) (Table 2). Linoleic acid content varied from 55.8% belonging to Mex.22-191 to 75.5% belonging to A_2_ genotype (Table 2). The ranges of 2–4%, 6–8%, 16–20% and 70–75% for stearic acid, palmitic acid, oleic acid and linoleic acid for safflower cultivars have been reported by other workers [32–34]. These discrepancies in protein, oil and fatty acid composition of seed of the safflower genotypes reported here and elsewhere were mainly due to the genes and environmental effects that could jointly influence these traits in safflower [4]. Further research to resolve these discrepancies is needed to understand the direction of gene effects on the one hand, and the knowledge of interplay between the genes and environments on the other.

Seed ash content of the genotypes ranged from 3.10% belonging to ISF_28_ to 0.92% belonging to Mex.13-216 (Table 2). The safflower genotypes also varied significantly for fiber content ranging from 33% in Mex.17–45 to 42.4% in K_21_ (Table 2). The high variation observed for the biochemical traits of the seed of safflower genotypes could be attributed to the influences of these traits by genotype, environment and their interactions [33].

Calculation of genetic coefficient of variation revealed that the highest and the lowest of genetic variations belong to seed ash and fiber content, respectively (Table 2). Based on phenotypic coefficient of variation, the highest and the lowest values belonged to oleic acid content (%) and fiber content (%), respectively (Table 2). This result showed that fiber content is the least environmentally affected seed quality trait.

Pearson correlation coefficient was calculated for the seed quality-related traits (Table 3). The highest negative relationship was observed between oleic acid and linoleic acid content (*r* = −0.98 **). The negative association between oleic acid and linoleic acid was also reported in safflower by Fernandez-Martinez *et al.* [32], and Mahasi *et al.* [29]. A negative significant correlation was observed between oil content and stearic acid (*r* = −0.50 *). This result is in agreement with that of Johnson *et al.* [34], who observed a significant and negative correlation between stearic acid and oil content (%) in safflower genotypes. Palmitic acid correlated with oleic acid significantly and negatively (*r* = −0.51 *). A positive significant correlation (*r* = 0.54 *) was observed between protein content and palmitic acid. Strong positive relationship was found between protein content and ash content (*r* = 0.66 **). Protein content and fiber content was also correlated significantly (*r* = 0.54 *). Revealing the relationships between seed quality-related traits could help in planning effective breeding strategies for simultaneous improvement of these traits in safflower.

**Figure 1.**
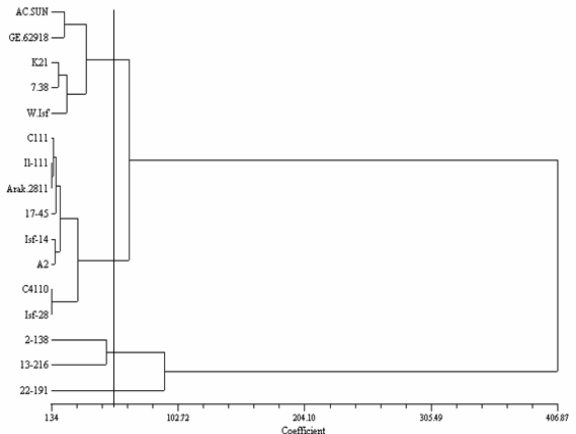
UPGMA-based dendrogram showing genetic relationship among 16 safflower genotypes based on seed quality-related traits.

Clustering based on seed quality-related traits divided the genotypes into four groups (Figure 1). Group 1 includes GE_62918_ from Germany, AC-Sunset from Canada, Mex.7–38 from Mexico and two Iranian lines (K_21_ and Wht-ISF). Group 2 was the largest group and includes 5 Iranian lines (IL.111, Arak-2811, ISF_14_ and A_2_) and a Mexican cultivar (Mex.17-45). Group 3 only possesses two Mexican cultivars (Mex. 2-138 and Mex.13-216). Group 4 includes a Mexican cultivar (Mex.22-191).

There was a broad genetic variation among safflower genotypes for fatty acids, oil and protein content. These variations in the seed quality-related traits imply a considerable potential of the studied genotypes for safflower improvement. Also, the present study showed oleic type genotypes including Mex.7-38 and Mex.22-191 (Table 3). An Iranian line (A_2_) originated from a cold climate of Iran (Azarbayjan province) has an elevated linoleic content (75.5%). This finding might be related to the impact of temperature and geographical origin in the diversity of fatty acid contents [33]. Genetic variation for fatty acids and biochemical traits was also reported in safflower by other studies [8,22,34]. Variation of unsaturated fatty acids in safflower oil points out the possibility of improving oil quality through the breeding programs [10]. Although, genotypes from different geographical regions clustered in a same group (Figure 1), two Mexican genotypes created a separate cluster. Moreover, Mex.22-191, as an oleic type genotype, separated from the other genotypes. Considering the results of cluster analysis, genotypes from distant clusters, could be used to produce superior hybrid with elevated seed quality-related traits. In this study, a clear relationship between diversity patterns and geographical origin was not revealed. The lack of relationship of the genotypic clusters for seed quality-related traits with the geographical origins was also reported by others [11].

### 2.2. ISSR Analysis

**Figure 2.**
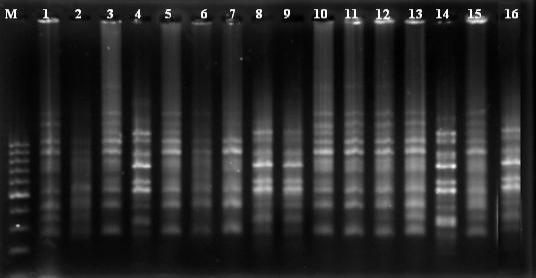
ISSR agarose gel profile of 16 safflower genotypes using primer number 16 (as described in Table 2). Numbers on the top of wells correspond to the genotypic number listed in Table 6.

Out of 30 ISSR primers used, 20 showed polymorphism. Figure 2 shows the polymorphic fingerprinting pattern of the 16 safflower genotypes generated by ISSR primer number 16.

**Figure 3.**
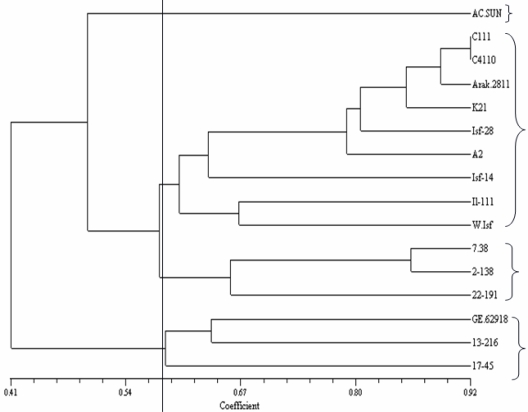
UPGMA-based dendrogram showing genetic relationship among 16 safflower genotypes based on Jaccard’ s similarity estimates obtained for ISSR markers.

A total of 204 bands were scored for 20 ISSR primers (Table 4). With an average of 10.2 bands per primer, a total of 204 bands generated and 149 bands (73%) of these were polymorphic. Total generated band ranged from 5 to 16 and the polymorphic bands of the primers ranged from 3 to14 bands. The mean of polymorphic bands for (GA)*_n_*, (AC)*_n_*, (AG)*_n_*, (CT)*_n_*, (TCC)_5_, (TCC)_7_ and (TG)*_n_* primers was 12, 11, 10.4, 9.3, 8.5 and 8, respectively. The mean of polymorphism for (CT)*_n_*, (AC)*_n_*, (TCC)_7_, (TCC)_5_ and (TG)*_n_* was 83%, 76.5%, 69.5%, 58.66% and 50%, respectively. Two motif primers of (CT)*_n_*, (AC)*_n_* and (AG)*_n_* types produced the highest mean of polymorphism. The un-weighted pair-group method with arithmetic mean (UPGMA) cluster analysis showed that 16 safflower genotypes were grouped into four marker-based groups (Figure 3). Cluster 1 comprises AC-Sunset, a Canadian genotype. Cluster 2 includes 9 Iranian lines from different geographical regions that compromised from Northern, Western and Central regions of Iran. Cluster 3 possessed 3 Mexican genotypes includes Mex.7-38, Mex.2-138 and Mex.22-191. Finally, cluster 4 contains 2 Mexican genotypes (Mex.13-216 and Mex.17-45) accompanied with GE_62918_ from Germany.

Principal coordinate analysis (PCoA) for displaying the relationships among the safflower genotypes was performed. The first three principal coordinates explained 75.56% of the total variation, with 59.82% explained by the first, 9.48% by the second and 6.25 by the third at the DNA sequence level (Table 5).

The ISSR primers used in this study revealed an acceptable genomic variation among the selected genotypes. The relationships between the genetic distances estimated based on seed quality traits and molecular markers was not statistically significant (*r* = 0.13).

Analysis of molecular variance (AMOVA) revealed a non-significant difference between two groups of genotypes (native *vs.* exotic). AMOVA for ISSR data also indicated significant variations within the genotypic groups.

Similar to seed quality-related traits, high molecular genetic variation was observed which is in agreement with the observation of high efficiency of ISSR marker in detecting genetic variation in safflower by Yang *et al.* [27]. Assessment of genetic diversity, based on both molecular markers and seed quality-related traits including biochemical traits and fatty acid composition, has only been employed in a few tree species [12,13].

In this study, the most polymorphic bands was related to (CT)*_n_*, (AC)*_n_*, and (AG)*_n_* primers. Similar to our results, Yang *et al.* [27], has reported that (AC)*_n_* and (CT)*_n_* ISSR primers produced the highest polymorphic bands in safflower. In this study, (GT)*_n_* primers have not produced any band, this finding was in agreement with Yang *et al.* [27]. On the other hand, in rice Blair *et al.* [15], have reported that (GT)*_n_* primers have produced a high frequency of banding pattern because of additional nucleotide in 5′ terminal sequence. Because of the high frequency of polymorphic band generated by (GA)*_n_*, (CT)*_n_* and (AC)*_n_* primers, it can be deduced that these motifs have a significant contribution in the safflower genome.

The cluster analysis based on ISSR data showed that there was a considerable agreement between geographic origin and *their* genomic similarities. However, in viewpoint of molecular clustering, similarities in genotypes that grouped in the same cluster could also arise because of sharing a common parentage, convergent evolution and selection. According to Figure 3, GE_62918_ (from Germany) and AC-Sunset (from Canada) have clustered in a same group with two Mexican cultivars. They could probably have originated from a common ancestor or probability of being duplicates [11]. A non-significant correlation between seed quality-related traits and ISSR markers in the Mantel test [35] suggested there was a slight similarity between these clusterings. This could be due to the exchange of plant material across the regions during the evolution of safflower [11]. Another reason could be that ISSR markers detect polymorphism in coding and non-coding regions of genome, but seed quality-related traits are the results of expressed sections of the genome [11].

Although some work based on morphological traits has been conducted to assess genetic variation in safflower genotypes, the results are still ambiguous, because phenotypic traits are affected by developmental stages and environmental conditions. On the other hand, ISSR markers overcome those disadvantages to a certain extent. The results of the present study are consistent with those of Yang *et al.* [27], who emphasized that ISSR, is an effective marker system for detecting genetic diversity among safflower genotypes and provides useful information about the phylogenic relationships.

## 3. Materials and Methods

### 3.1. Plant Materials and Growth Conditions

Sixteen safflower (C. *tinctorious*) genotypes including 9 with native and 7 with exotic origins were used (Table 6). Field experiment was conducted at Research Farm of Isfahan University of Technology, Isfahan, Iran (51°32′ E and 32°32′ N, 1630 m) in 2008. A randomized complete block design with three replications was used. Each plot consisted of three rows 40 cm apart and 5.5 m in length. Fertilizers were applied prior to sowing at a rate of 50 kg N ha^−1^ and 30 kg P ha^−1^, and additional side dressing of 50 kg N ha^−1^ was applied at the early flowering stage.

### 3.2. Oil Extraction

Seeds of the entries were dried at 60 °C for 4 h, using a ventilated oven, up to a moisture content of about 5%, and were then ground with a blender. Ten grams of ground seeds were used to extract the oil, using petroleum ether for 6 h in a Soxhlet system according to the American Oil Chemists’ Society (AOCS) method [36], and then the oil content as a percentage was calculated for each sample.

### 3.3. Fatty Acid Composition

The fatty acid composition of the oil samples was determined by gas chromatography (AOCS Ce 1–62; AOCS 1993) method. The oil sample of each experimental unit (plot) was converted to its fatty acid methyl esters (FAME). Oil samples (0.2 mL) were dissolved in hexane and transesterified with sodium methylate (0.1 M). Analyzes of FAMEs were carried out using an Agilent 6890 model gas chromatograph (Agilent Technologies, Palo Alto, CA, USA) equipped with a with a split-injection port, flame ionizing detector (FID) and a fused silica capillary column (HP-88, 100 m × 0.25 mm i.d.; film thickness = 0.2 μm). The samples (1.0 μL) were injected in split mode (split ratio 1:50). The initial oven temperature was set at 150 °C for 1 min, elevated at a rate of 5 °C min^−1^ to 190 °C for 2 min, and then ramped at 5 °C min^−1^ to the final 240 °C for 8 min. The injector temperature was set at 250 °C and the detector temperature was set at 280 °C. Nitrogen with at a flow rate of 1.5 mL min^−1^ was used as the carrier gas. Peak identification was performed by comparing the relative retention times with those of a commercial standard mixture of FAME. The fatty acid content of palmitic (C16:0), stearic (C18:0), oleic (C18:1), linoleic (C18:2) and linolenic (C18:3) were determined using a computing integrator and showed as the percentage of the oil.

The fatty acid content of myristic (C14:0), palmitic (C16:0), stearic (C18:0), oleic (C18:1), linoleic (C18:2), and linolenic (18:3) were determined. Only the trace amount of myristic and linolenic acids were detected and hence were not further considered. The relative percentage of each fatty acid was determined by integration of each peak in the chromatogram.

### 3.4. Seed Protein, Fiber and Ash Contents

Protein (%), fiber (%) and ash (%) contents of seeds from each sample were estimated using near-infrared reflectance spectroscopy (NIR) (model 8200, Perten Instruments AB, Sweden). Forty-eight samples (three field replications of the genotypes) were scanned three times.

### 3.5. ISSR Analysis

Genomic DNA was extracted from 25–30 plants from young leaves of each genotype following the protocol of Murray and Thompson [37]. DNA was quantified electrophoretically using lambda standard DNA on 0.7% agarose gel. Thirty ISSR primers were used in this study. PCR reaction was performed using 15 μL PCR mixtures containing 1.5 μL of 10× buffer (0.1 M of Tris-HCL, pH 8.3, 0.5 m KCl), 2 Mm MgCl2, 200 μM dNTPs, 2 μL each primer (10 pM concentration), 2 μL of DNA template (30 ng) and 1 U *Taq* DNA polymerase. From a total of 30 primers used, 20 capable of producing repeatable and polymorphic bands were used for PCR amplification (Table 1), using Peqlab Primus 96 advanced thermal cycler (Peqlab, Erlangen, Germany). DNA thermal cycler was programmed by 40 cycles of 1 min at 94 °C, 1 min at particular annealing temperature for each primer and 2 min at 72 °C, then a final extension of 7 min at 72 °C. The products of amplified PCR were separated by electrophoresis on 1.4% agarose gels using gel electrophoresis equipped with Biometra Model PS9009TC power supply at 50 W for 2 h in 1× TBE buffer.

Gels were stained with ethidium bromide (0.5 mg/mL). DNA banding patterns were visualized using Biometra gel documentation Model S2.

The ISSR bands were scored as presence (1) or absence (0) for each of 16 genotypes with the 20 primers. The binary matrix was subjected to statistical analyses by NTSYS-pc, version 2.5 [38]. Jaccard’s similarity coefficient was employed to compute pair wise genetic similarities. The corresponding dendrogram was constructed by applying un-weighted pair-group method with arithmetic mean (UPGMA). Number of polymorphic bands and percentage of polymorphism were calculated for each primer.

For achieving the association between seed quality traits and molecular distances of genotypes, the relationships between two matrices was assessed using the Mantel test [35]. Principal coordinate analysis (PCoA) was performed for showing the relationships among the genotypes [38]. Analysis of molecular variance (AMOVA) was performed to estimate variance components for ISSR data, partitioning the variation into within and among two types of genotypic origins (exotic and native) using Arliquin 2.0 software [39].

### 3.6. Statistical Analysis

The analysis of variance (ANOVA) of data for seed quality-related traits was performed using General Linear Model of SAS program [40]. Mean comparisons were conducted using the Fisher’s least significant difference (LSD_0.05_) test. Clustering of genotypes based on seed quality-related traits and molecular markers was carried out using UPGMA method [38]. The means of each trait were used for cluster analysis. Euclidean distance was used for cluster analysis with the unweighted pair group arithmetic means method (UPGMA) by using NTSYS-pc software version 2.02. PCoA was carried out with NTSYS_pc statistical package, version 2.02 [38]. Cluster and principal coordinate analysis were performed to assess patterns of diversity among genotypes and to select the most distant genotypes from each group based on molecular markers.

## 4. Conclusions

This is the first published report on the assessment of genetic diversity in a field crop using both molecular markers and seed quality-related traits. In summary, our results showed significant variation among the genotypes of safflower for most of the biochemical traits studied. The findings of high genetic variation for both seed quality-related traits and polymorphism at DNA level reveal that seed quality traits can be efficiently improved by the selection programs in safflower. Cluster analysis based on ISSR markers divided the genotypes into four distinct groups possessing logical similarity with their geographical origin.

## Figures and Tables

**Table 1.** Analysis of variance for seed quality-related traits in safflower genotypes.

**Source of variation**		**Mean Squares**							
	**df**	**C16:0^†^**	**C18:0**	**C18:1**	**C18:2**	**Oil (%)**	**Protein (%)**	**Ash (%)**	**Fiber (%)**
Replication	2	0.89 *	0.25 **	34.41	89.91	1.06	3.06 *	0.001	0.47
Genotype	15	5.09 **	1.31 **	155.7 **	196.6 **	31.69 **	38.07 **	1.40 **	22.16 **
Residual	30	0.23	0.03	45.62	90.09	0.75	0.707	0.14	2.00

* and ** significant at *P* < 0.05 and *P* < 0.01, respectively.

† Fatty acids: palmitic (C16:0), stearic (C18:0), oleic (C18:1), linoleic (C18:2).

**Table 2.** Mean comparisons of the seed quality-related traits in safflower genotypes.

**Genotype**	**C16:0 ^†^**	**C18:0**	**C18:1**	**C18:2**	**Oil (%)**	**Protein (%)**	**Ash (%)**	**Fiber (%)**
AC-Sunset	7.90	1.52	19.50	71.56	29.36	22.80	2.70	39.83
GE_62918_	7.29	2.94	18.20	70.56	25.54	20.10	1.80	41.10
C_111_	9.61	2.60	15.95	72.61	27.37	18.50	2.51	41.40
C_4110_	10.37	2.03	15.42	72.11	27.65	23.70	2.80	39.70
ISF_14_	11.07	1.50	14.1	73.96	29.72	25.46	1.80	35.00
A_2_	8.20	2.57	13.78	75.54	26.50	23.30	2.71	39.00
K_21_	8.83	2.90	20.86	67.80	30.62	25.60	2.50	42.36
ISF_28_	10.52	2.30	15.5	71.58	31.06	20.30	3.10	37.40
IL.111	9.08	1.81	15.64	73.45	25.08	18.01	1.93	38.83
Wht-ISF	8.43	2.80	22.79	65.71	20.95	19.10	1.75	35.30
Arak-2811	7.70	2.04	14.97	75.43	27.30	18.40	2.4	37.3
Mex.7-38	7.96	2.93	35.28	53.83	26.81	21.30	2.64	38.50
Mex.2-138	9.47	1.56	26.59	62.37	33.50	22.40	2.30	39.03
Mex.22-191	7.30	1.86	35.26	55.82	32.65	17.55	1.03	34.80
Mex.13-216	6.49	1.43	29.42	62.66	31.50	13.50	0.92	35.02
Mex.17-45	8.30	2.40	18.77	70.83	31	14.30	1.00	33.01
LSD_0.01_	1.08	0.41	15.16	21.3	1.95	1.88	0.85	3.17
Genotypic variance	1.62	0.42	36.68	35.5	10.31	12.45	0.42	6.72
Phenotypic variance	1.69	0.43	51.88	65.5	10.56	12.68	0.46	7.38
Genotypic CV (%)	14.5	24.5	29.15	8.71	11.24	17.36	30.33	6.82
Phenotypic CV (%)	14.8	24.9	34.69	11.8	11.35	17.26	31.75	7.13

† Fatty acids: palmitic (C16:0), stearic (C18:0), oleic (C18:1), linoleic (C18:2).

**Table 3.** Correlation coefficients for seed quality-related traits of 16 safflower genotypes.

	**^†^C16:0**	**C18:0**	**C18:1**	**C18:2**	**Oil (%)**	**Protein (%)**	**Ash (%)**	**Fiber (%)**
C16:0	1							
C18:0	−0.17	1						
C18:1	−0.51 *	−0.02	1					
C18:2	0.39	−0.05	−0.98 **	1				
Oil	0.05	−0.50 *	0.29	−0.26	1			
Protein	0.54 *	0.09	−0.27	0.19	−0.03	1		
Ash	0.48 *	0.23	−0.39	0.31	−0.16	0.66 **	1	
Fiber	0.13	0.34	−0.25	0.21	−0.17	0.54 **	0.67 **	1

* and ** significant at *P* < 0.05 and *P* < 0.01, respectively.

† Fatty acids: palmitic (C16:0), stearic (C18:0), oleic (C18:1), linoleic (C18:2).

**Table 4.** ISSR primers, number of fragments, number of polymorphic fragments and percentage of polymorphism generated in the safflower genotypes.

**Primer**	**Sequences**	**Total Number of Fragments**	**Number of Polymorphic Fragments**	**Polymor-Phism (%)**
1	3′-C(AG)_8_-5′	16	14	87
2	3′-(CCT)_7_H*V*H*-5′	9	7	77
3	3′-YC(AG)_8_-5′	11	9	81
4	3′-C(CA)_8_-5′	10	7	70
5	3′-GY(CA)8-5′	12	11	91
6	3′-CY(GA)8-5′	13	11	84
7	3′-GY(GA)_8_-5′	9	7	78
8	3′-YR-(CCT)_5_-5′	11	9	81
9	3′-(CCT)_5_H*V*H-5′	8	5	62
10	3′-GR(TC)_8_-5′	12	11	91
11	3′-(CCT)_7_B*D*B*-5′	8	5	62
12	3′-(CA)_7_D*B*D*-5′	9	5	55
13	3′-GY(CA)_8_-5′	11	10	90
14	3′-(CCT)_5_D*B*D*-5′	9	3	33
15	3′-C(GA)_8_-5′	5	3	60
16	3′-TY(GA)_8_-5′	14	12	85
17	3′-Y(AG)_8_-5′	12	3	25
18	3′-G(TC)_8_-5′	8	6	75
19	3′-GY(AG)_8_-5′	9	7	77
20	3′-TR(GT)_8_-5′	8	4	50
Total		204	149	73
Average		10.2	7.45	

R = A/T, Y = G/C, B = T/G/C, D = A/T/G, H = A/T/C, V = 3A/G/C.

**Table 5.** Eigenvalue, explained variance and cumulative in the principal coordinate analysis (PCoA) used to classify 16 safflower genotypes by ISSR markers.

**Principal Coordinate**	**Eigenvalue**	**Explained Variance (%)**	**Cumulative Variance (%)**
1	9.57	59.82	59.82
2	1.51	9.48	69.30
3	1.01	6.25	75.56

**Table 6.** Safflower genotypes used in this study.

**Entry**	**Genotype**	**Origin**
1	C_111_	Selected line from Kouse landrace
2	C_4110_	Selected line from Kouse landrace
3	ISF_14_	Selected line from Isfahan landrace
4	A_2_	Selected line from Azarbayjan landrace
5	K_21_	Selected line from Kordestan landrace
6	ISF_28_	Selected line from Isfahan landrace
7	IL.111	Selected line from Auromyeh landrace
8	Wht-ISF	Selected line from Isfahan landrace
9	Arak-2811	Selected line from Markazy landrace
10	AC-Sunset	Canada
11	GE_62918_	Germany
12	Mex.7-38	Mexico
13	Mex.2-138	Mexico
14	Mex.22-191	Mexico
15	Mex.13-216	Mexico
16	Mex.17-45	Mexico
